# Dietary Patterns of Patients with Chronic Kidney Disease: The Influence of Treatment Modality

**DOI:** 10.3390/nu11081920

**Published:** 2019-08-15

**Authors:** Fernanda Santin, Daniela Canella, Camila Borges, Bengt Lindholm, Carla Maria Avesani

**Affiliations:** 1 Graduate Program in Food, Nutrition and Health, Nutrition Institute, Rio de Janeiro State University, Rio de Janeiro 20559-900, Brazil; 2 Department of Applied Nutrition, Nutrition Institute, Rio de Janeiro State University, Rio de Janeiro 20559-900, Brazil; 3 Department of Nutrition, School of Public Health, University of São Paulo, São Paulo 01246-904, Brazil; 4 Division of Renal Medicine and Baxter Novum, Department of Clinical Science, Intervention and Technology, Karolinska Institute, 14186 Stockholm, Sweden

**Keywords:** dietary patterns, chronic kidney disease, exploratory factor analysis, unhealthy dietary pattern, healthy dietary pattern

## Abstract

Background: We analyzed the dietary patterns of Brazilian individuals with a self-declared diagnosis of chronic kidney disease (CKD) and explored associations with treatment modality. Methods: Weekly consumption of 14 food intake markers was analyzed in 839 individuals from the 2013 Brazil National Health Survey with a self-declared diagnosis of CKD undergoing nondialysis (*n* = 480), dialysis (*n* = 48), or renal transplant (*n* = 17) treatment or no CKD treatment (*n* = 294). Dietary patterns were derived by exploratory factor analysis of food intake groups. Multiple linear regression models, adjusted by sociodemographic and geographical variables, were used to evaluate possible differences in dietary pattern scores between different CKD treatment groups. Results: Two food patterns were identified: an “Unhealthy” pattern (red meat, sweet sugar beverages, alcoholic beverages, and sweets and a negative loading of chicken, excessive salt, and fish) and a “Healthy” pattern (raw and cooked vegetables, fruits, fresh fruit juice, and milk). The Unhealthy pattern was inversely associated with nondialysis and dialysis treatment (β: −0.20 (95% CI: −0.33; −0.06) and β: −0.80 (−1.16; −0.45), respectively) and the Healthy pattern was positively associated with renal transplant treatment (β: 0.32 (0.03; 0.62)). Conclusions: Two dietary patterns were identified in Brazilian CKD individuals and these patterns were linked to CKD treatment modality.

## 1. Introduction

Chronic kidney disease (CKD) is a common noncommunicable disease associated with high morbimortality and has an estimated worldwide prevalence of 11%–13% among adults [1]; in Brazil, the prevalence is approximately 9%, not including patients on dialysis [2]. Early diagnosis, adequate pharmaceutical treatment, and lifestyle changes—with dietary modifications adapted to the stage of CKD and treatment modality (nondialysis, dialysis, and renal transplantation)—are important interventions for reducing the progression of the disease and cardiovascular morbidity and mortality [3]. Cardiovascular mortality has been shown to be 30 times higher among people with end-stage kidney disease (ESKD) than in the general population [4]. Among factors contributing to the abysmal clinical outcomes in these patients, poor adherence to prescribed pharmacologic and nonpharmacologic treatments is a common albeit potentially modifiable problem, not least of which involves adherence to dietary advice provided by dietary counseling, which is one of the cornerstones of nonpharmacologic care for CKD patients [5,6,7,8,9].

The crucial role of nutritional interventions in preventing or retarding the rate of progression of CKD and its complications is well established [10,11]. However, most studies and nutritional guidelines addressing the care of individuals with CKD have focused primarily on dietary recommendations regarding the intake of macronutrients (i.e., energy and protein intake) and the restriction of single micronutrients, such as sodium, potassium, and phosphorus [12,13,14,15,16], while usually not considering dietary patterns reflecting the overall quality of the diet, which conceivably may play an even more important role in clinical outcomes. The analysis of dietary patterns has emerged as a practical approach to evaluate qualitative as well as quantitative aspects of the overall diet, an approach which considers the simultaneous effect of multiple foods and dietetic components, as well as their interactions [17].

Dietary patterns can be derived by a priori and a posteriori analyses. The choice of method depends on the purpose of the study. In the a priori approach, indices are proposed, which allow the evaluation of the diet quality based on pre-existing conceptual criteria that form the basis for guidelines for healthy eating and specific nutritional recommendations [17,18]. The Healthy Eating Index and Mediterranean Diet Score are examples of indices used to evaluate overall diet quality. In the a posteriori approach, the dietary pattern is based on the actual food intake of the population that is evaluated. This exploratory method uses multivariate techniques, such as exploratory factorial analysis, principal component analysis, and cluster analysis, to derive food patterns [17,19]. The great majority of the studies that have evaluated the dietary pattern in individuals with CKD have used the a priori method [20,21,22,23]; however, a posteriori approaches have the advantage of not making assumptions about diet quality based upon diet–disease associations but rather describe dietary patterns based on foods often consumed in the studied population. To our knowledge, there is no population-based study evaluating the dietary patterns of CKD patients derived by the a posteriori method.

The aim of this study was to describe the dietary patterns of a population of Brazilian individuals from a national survey that included participants with a self-declared CKD diagnosis. As we have reported previously, the consumption of food items normally restricted to CKD patients differs from that of non-CKD individuals, and the treatment modality influences the type and food consumption frequency of CKD patients [24]. Thus, in the current study, we extended our analysis by testing the hypothesis that CKD treatment modality (conservative, dialysis, or renal transplantation) or lack of regular treatment (neither conservative, dialysis, nor renal transplantation) influences dietary patterns.

## 2. Materials and Methods

### 2.1. Study Population and Sampling

This was a nationally representative cross-sectional study using data from the National Health Survey (Brazilian NHS) 2013 involving the Brazilian adult (≥18 years) population. The Brazilian NHS was conducted in partnership between the Ministry of Health and the Brazilian Institute of Geography and Statistics (IBGE) and its main objective was to produce nation-level data about the health status and lifestyles of the Brazilian population [25].

The Brazilian NHS sample was a subsample of the Master Sample of the Integrated Household Surveys System of the IBGE with stratified sampling and three clustering stages: census tracts, households, and individuals who were ≥18 years selected by a random sample of residents in each household. A total of 60,202 individuals were interviewed. The complete information regarding the process of sampling and weighting is available in a prior publication [26]. The current study comprised self-declared participants with a medical diagnosis of chronic kidney disease (*n* = 839 individuals).

The Brazilian Ministry of Health’s National Commission of Ethics in Research approved the Brazilian NHS under protocol number 328,159 (June 26, 2013).

### 2.2. Data Collection and Variables of Study

Interviews were conducted from August 2013 to February 2014. The questionnaire was applied through face-to-face interviews and it was subdivided into three parts, related to: household, all the household residents, and individuals. The present study used data from the individual questionnaire and data related to all the household residents.

The Brazilian NHS investigated the food consumption frequency of the adults through the weekly consumption of markers of food intake or behaviors related to intake [27,28]. The use of food intake markers was validated by previous studies [29,30,31] using data from the Brazilian surveillance system for risk factors of chronic diseases (VIGITEL). In these studies, the validity was analyzed by comparing the results of telephone interviews (using food intake markers) with results of three 24 h recalls (considered as a gold standard) carried out up to five days following the original interview [29,30,31]. The food intake markers evaluated in the present study were fruit, fresh juice fruit, vegetables (raw or cooked), beans, milk, sugar-sweetened beverages (SSB), red meat (pork, veal, beef, or lamb), chicken, fish, replacement of meals with snacks, excess salt, and alcoholic beverages (Table 1).

This study also considered the following sociodemographic variables—gender, age, education level (no primary school, incomplete primary school, complete primary school but incomplete secondary school, complete secondary school, and incomplete or complete university), and race/skin color (white, black, yellow, mixed race, and indigenous)—and geographical variables—location of residence (urban and rural) and Brazilian geographical regions (North, Northeast, Southeast, South, and Midwest).

For the current study, participants with a self-declared CKD diagnosis were grouped according to their treatment in the following categories: nondialysis-dependent group (individuals who were not on dialysis), dialysis-dependent group (individuals on hemodialysis or peritoneal dialysis), untreated CKD group (individuals who declared not to be under regular medical treatment—neither conservative management nor dialysis), and renal transplanted group (individuals who declared that they received a renal transplant). Self-reported anthropometric measurements (body weight and height) and self-reported presence of comorbidities (diabetes, hypertension, and cardiovascular disease) were used to characterize the population.

### 2.3. Statistical Analysis

For descriptive purposes, the values are presented as mean and 95% confidence intervals (95% CI) for continuous variables and as weighted percentages for categorical variables. Dietary patterns for 14 food intake markers were obtained by exploratory factor analysis (based on principal component factor). Kaiser–Meyer–Olkin (KMO) over 0.60 [32] and Bartlett’s sphericity test (BTS) with a *p*-value lower than 0.05 [33] were used to assess sample adequacy and to check the applicability of the data for factor analysis. The number of factors to be retained in the exploratory factor analysis was selected by eigenvalues over 1.5; the Cattel test graph (screen plot), for which values located before the inflection point line indicated the number of factors to be retained; and the factor loading interpretability. After the choice of number of factors, the varimax rotation was executed to maximize higher factor loadings and to minimize the lowers, assuring a better factorial loading distribution and simplifying the interpretation. The food groups with factor loadings (according to analysis with rotation) │0.35│ were considered representative of that pattern. Positive factor loadings (>0.35) showed positive correlations between the food group and the dietary pattern, while negative factor loadings (<-0.35) showed negative correlations. The communalities were also evaluated, and a minimum cutoff of 0.20 was considered acceptable for each food item in the model. Dietary patterns were named according to the dominant foods in the respective patterns and their interpretability. Factor scores of the dietary patterns were estimated for each individual, which were used in subsequent analyses. A higher score indicated higher adherence to the respective pattern.

Linear regression models (crude and adjusted by gender, age, educational level, race/skin color, location of residence, and Brazilian geographical regions) were applied to assess whether there were differences in the dietary pattern scores between the CKD treatment groups as compared to the untreated CKD group (reference group). Negative score means indicated an inverse association and positive score means indicated a positive association. All analyses were performed using Stata software version 14.2, which considered a level of significance of 5% and the effects of complex sampling from the Brazilian NHS (survey module).

## 3. Results

Among the 60,202 individuals interviewed in the Brazilian NHS, 839 individuals (1.4%; 95% CI 1.3; 1.6%) self-reported a medical diagnosis of CKD and were included in this study. Out of 839 individuals, 57% (*n* = 480) were not on dialysis (nondialysis-dependent group), 6% (*n* = 48) were on dialysis (dialysis group), 2% (*n* = 17) underwent kidney transplantation (renal transplanted group), and the remaining 35% (*n* = 294) declared not to be under regular medical treatment (neither conservative management nor dialysis—untreated CKD group). Table 2 shows the main characteristics of the participants.

Table 3 shows the mean and prevalence of consumption frequency of food intake markers in individuals with CKD. It is noteworthy that a high proportion of the CKD individuals reported never consuming juice fruit, sweets, sugar-sweetened beverages, alcoholic beverages, and meal-replacement snacks; the consumption of fish and chicken one to two times a week; and daily consumption of milk, fruit, vegetables, and beans. Regarding the perception of salt intake, 48% of the participants considered their salt intake appropriate.

Based on the Cattel test graph (screen plot) (Figure S1), eigenvalues of >1.5, and considering the interpretability of the patterns, two factors were retained, explaining 32.1% of the variability of consumption (Table 4). The first pattern, named the “Unhealthy” pattern, explained 18.3% of the total variance and consisted of consumption of red meat, sweet sugar beverages, alcoholic beverages, and sweets and a negative loading of chicken, excessive salt, and fish. In contrast, the second pattern, named the “Healthy” pattern, explained 13.8% of the total variance and was characterized by high factor loadings for a combination of fresh foods, raw and cooked vegetables, fruits, fresh fruit juice, and milk. The food consumption markers of beans and replacement of meals with snacks were excluded as their communalities were <0.20. On the other hand, the consumption of alcoholic beverages, sweets, and milk also presented communalities of <0.20 but were kept in the model since they allowed for discrimination of the food patterns.

Table 5 shows the β-coefficients (95% CI) between CKD treatment and mean scores of dietary patterns. After adjusting it by sociodemographic and geographical variables and comparing it with the untreated CKD group (reference category), the nondialysis-dependent and dialysis-dependent groups had an inverse association with the Unhealthy pattern (β: −0.20 (95% CI: −0.33; −0.06) and β: −0.80 (95% CI: −1.16; −0.45), respectively) meaning that these groups had low adherence to the Unhealthy pattern. In addition, the renal transplant group had a positive association (β: 0.32 (95% CI: 0.03; 0.62)) with the Healthy pattern, suggesting that they were more often adhering to this pattern, as compared with the untreated CKD group.

## 4. Discussion

This study analyzed dietary patterns in a sample of the Brazilian NHS comprising Brazilian individuals who had a self-declared diagnosis of CKD and the association of CKD treatment modality with dietary patterns. Two dietary patterns, labeled Unhealthy and Healthy, were identified in the whole group. In a previous study comprising Brazilian nondialyzed patients with CKD stages 3 and 4 (*n* = 454), three dietary patterns were found: snack pattern (breads, biscuits, cakes, farinaceous products, butter, margarine, eggs, processed meat, sweets, snacks, whole dairy products, and sweetened beverages); *mixed pattern*, consisting of healthy foods but with red meat (whole grains, pasta, tubers, red meat, poultry, fish, seafood, fruits, vegetables, low-fat dairy products, and natural juice); and *traditional pattern* (white rice, beans, and coffee) [34]. The findings from the present study and our previous study underline the diversity of food patterns in CKD patients. Of note, Machado et al. [34] also demonstrated that gender, school degree, and comorbidities, such as diabetes and hypertension, influenced dietary patterns. The latter findings are in accordance with those reported in our previous study—based on the same Brazilian NHS survey—in which the sociodemographic and geographical variables were found to influence the food consumption frequency in CKD patients [24].

Moreover, when exploring the influence of the CKD treatment, we observed that, compared to the untreated CKD group, the nondialysis-dependent and dialysis groups had an inverse association with the Unhealthy dietary pattern, while the renal transplanted group had a positive association with the Healthy dietary pattern. Of interest is the finding that the transplanted group adhered to a diet with better dietary quality. We speculate that this may suggest that renal transplanted patients, to a large extent, represent individuals under close medical follow-up who are highly motivated to follow medical prescriptions as well as prescriptions regarding nonpharmacological care, such as a healthier diet, since nonadherence to given prescriptions is associated with increased risk of graft loss and mortality, as previously shown [35,36,37]. In addition, as renal transplanted patients are no longer advised to restrict the intake of food sources of potassium, such as fruits and vegetables, which were part of the Healthy dietary pattern in the current study, it is possible that the less restrictive dietary counseling influenced the adherence of renal transplanted patients to the Healthy dietary pattern.

Previous studies carried out in CKD individuals reported associations of dietary patterns with increased risk of CKD occurrence, loss of renal function, and death [38,39,40,41]. In a cross-sectional study of 1033 older Irish women, it was reported that an unhealthy dietary pattern was associated with lower renal function and greater prevalence of CKD [38]. In addition, Shi et al. [39] and Asghari et al. [40] showed that an unhealthy dietary pattern was positively associated with increased CKD prevalence, while a healthy dietary pattern was inversely associated with the occurrence CKD. Corroborating these findings, Rebholz et al. [41] observed an association of beverage patterns characterized by higher consumption of sugar-sweetened beverages with an elevated risk of CKD occurrence. Furthermore, Gutiérrez et al. [42] reported that a dietary pattern considered to be unhealthy was independently associated with increased risk of mortality, while a healthy pattern exerted a protective effect on the mortality risk [42]. Of note, Saglimbene et al. [43], by studying food consumption recorded as food groups in a multinational hemodialysis cohort, showed that a higher consumption of fruit and vegetables may reduce all-cause and noncardiovascular mortality [43]. Altogether, these studies suggest that unhealthy food patterns can lead to worse outcomes, including diminished renal function, the development of CKD, and higher mortality rates. However, to our knowledge, no prior study has specifically examined the association of CKD treatment modalities with dietary patterns. Our study addressed this gap in CKD individuals and indicated that CKD treatment may influence dietary patterns, although due to the cross-sectional study design, we cannot infer causality of CKD treatment over dietary patterns. This knowledge may inform dieticians and other healthcare providers about the need to consider treatment modality when giving dietary recommendations.

Some limitations of our study should be considered when interpreting these results. Due to the cross-sectional design, we cannot establish a causal relationship but can provide evidence by associations. Food intake markers, as well as other methods of assessing food intake, are limited tools that may not include all foods consumed. Although we adjusted for sociodemographic and geographical factors, we cannot exclude residual confounding from other lifestyle factors that may be linked with dietary habits and that partly could influence these associations. In addition, the small sample size and thus low statistical power of some groups may reduce the possibility of observing significant differences. Lastly, the presence of CKD and treatment modality were self-reported and there were no available laboratorial measurements of kidney function that would verify the presence or severity of CKD. Despite these limitations, the novelty of this study is that it describes dietary patterns of individuals with CKD receiving different treatment modalities. The use of exploratory factor analysis to assess food consumption should be highlighted, as it simultaneously analyzes multiple variables (different food groups), thus facilitating the description of food items and allowing assessment of the combined effect of foods commonly ingested by the Brazilian CKD population.

In conclusion, among Brazilian individuals with a self-reported diagnosis of CKD, two distinctly different dietary patterns associated with the modality of CKD treatment. The nondialysis-dependent and dialysis groups presented low adherence to the Unhealthy pattern, whereas individuals with a kidney transplant adhered to a better-quality diet. These findings may inform future recommendations about food and dietary patterns. While this new approach does not exclude the necessity of providing specific nutritional and energy recommendations, it adds a careful look at the quality of diets and addresses the principles and recommendations of healthy eating, in which the composition of the actual whole range of foods consumed comprises the principal dietary target.

## Figures and Tables

**Table 1:** Food intake markers evaluated in the study and determination of frequency of consumption.

Food Intake Markers	
Questions Used to Determine Frequency of Consumption	Food Items
“How many days a week do you usually eat/drink (name of food/beverage)? (a) ____ days (b) Never or less than once a week”	Fruit Fresh fruit juice Vegetables (raw or cooked) Beans Milk Sugar-sweetened beverages (soft drinks or artificial juice) Sweets Red meat (pork, veal, beef, or lamb) Chicken Fish Replacement of meals with snacks
“Considering freshly prepared food and industrialized food, do you think your salt intake is: (a) very high; (b) high; (c) appropriate; (d) low; (e) very low?”	Excess salt
“How often do you usually drink alcoholic beverages? (a) never; (b) once or more per month; (c) less than once a month”	Alcoholic beverages

**Table 2:** Main characteristics of participants with CKD from the Brazilian NHS, 2013 (*n* = 839).

Variables	All (*n* = 839)	Untreated CKD (*n* = 294)	Nondialysis Dependent (*n* = 480)	Dialysis Dependent (*n* = 48)	Renal Transplant (*n* = 17)
Sociodemographic Variables	Weighted % or Mean	Weighted % or Mean	Weighted % or Mean	Weighted % or Mean	Weighted % or Mean
Gender					
Male	44.9 (39.6; 50.2)	50.6 (41.7; 59.5)	36.9 (30.1; 44.3)	85.3 (72.2; 92.8)	48.7 (23.0; 75.2)
Age (in years)	53.5 (51.6; 55.4) *	53.6 (51.0;56.2) *	53.2 (50.4; 55.9) *	57.0 (49.7; 64.3) *	50.3 (43.1; 57.6) *
Education level					
No primary school	21.0 (16.8; 26.1)	19.2 (13.2; 26.9)	22.2 (16.5; 29.2)	26.7 (9.5; 55.8)	3.6 (0.9; 13.7)
Incomplete primary school	35.8 (31.0; 40.9)	34.7 (26.9; 43.5)	38.1 (31.5; 45.2)	27.1 (11.6; 51.4)	14.0 (3.2; 44.7)
Complete primary school but incomplete secondary school	12.6 (9.8; 16.2)	13.9 (9.2; 20.4)	10.4 (7.1; 15.0)	11.0 (4.2; 25.9)	61.5 (33.9; 83.3)
Complete secondary school	19.8 (16.1; 24.2)	22.4 (15.7; 30.9)	19.1 (14.2; 25.1)	13.1 (5.5; 27.9)	14.3 (3.3; 45.2)
Incomplete or complete university	10.8 (7.9; 14.3)	9.8 (5.3; 17.4)	10.2 (6.9; 14.7)	22.1 (9.1; 44.7)	6.6 (1.7; 22.5)
Race/Skin color					
White	52.7 (47.2; 58.2)	60.2 (52.0; 67.9)	49.8 (42.2; 57.5)	32.7 (16.7; 54.0)	62.4 (33.4; 84.6)
Black	9.6 (6.4; 14.1)	10.3 (6.1; 16.8)	9.3 (5.1; 16.2)	12.2 (2.4; 44.2)	0
Yellow	1.1 (0.4; 2.9)	1.0 (0.2; 3.9)	0.5 (0.2; 1.2)	8.9 (1.6; 38.0)	0
Mixed race	36.1 (31.1; 41.3)	28.5 (22.1; 35.9)	39.8 (32.9; 47.2)	44.2 (23.6; 66.9)	37.6 (15.4; 66.6)
Indigenous	0.5 (0.1; 2.0)	0	0.1 (0.0; 4.1)	2.0 (0.3; 13.2)	0
Location of residence					
Urban	86.5 (82.8; 89.4)	88.1 (82.3; 92.1)	86.0 (80.7; 90.0)	86.4 (67.6; 95.1)	72.1 (40.1; 90.9)
Rural	13.5 (10.6; 17.2)	11.9 (7.9; 17.7)	14.0 (10.0; 19.3)	13.6 (4.9; 32.4)	27.9 (9.1; 59.9)
Brazilian geographical regions					
North	6.2 (4.8; 8.0)	4.9 (3.4; 7.2)	7.5 (5.4; 10.4)	2.8 (1.0; 7.8)	0
Northeast	21.6 (17.5; 26.3)	14.7 (10.6; 20.1)	25.1 (19.0; 32.3)	28.6 (14.5; 48.7)	22.5 (7.8; 50.1)
Southeast	41.6 (35.9; 47.5)	45.3 (36.6; 54.4)	38.8 (31.1; 47.1)	37.4 (17.0; 63.5)	65.9 (39.1; 85.4)
South	22.3 (17.9; 27.4)	25.3 (18.4; 33.7)	21.1 (15.1; 28.7)	19.8 (7.7; 42.3)	8.3 (2.1; 27.9)
Midwest	8.4 (6.5; 10.8)	9.7 (6.3; 14.7)	7.4 (5.4; 10.2)	11.4 (3.9; 29.5)	3.2 (0.4; 20.5)
**Anthropometric Variables**	*n* = 836	*n* = 293	*n* = 478	*n* = 48	*n* = 17
Body weight (kg)	71.3 (69.7;73.0) *	74.2 (71.5; 77.0) *	69.5 (67.3; 71,8) *	71.2 (64.0; 78.4) *	71.9 (63.4; 80,4) *
Body mass index (kg/m^2^)	27.1 (26.5;27.6) *	27.5 (26.7; 28.4) *	26.9 (26.2; 27.6) *	25.5 (23.4; 27.6) *	27.2 (25.1; 29.2) *
**Comorbidity**					
Diabetes	*n* = 780	*n* = 242	*n* = 444	*n* = 45	*n* = 17
Yes	16.3 (12.7; 20.8)	16.0 (10.2; 24.3)	13.5 (9.6; 18.6)	37.7 (17.1; 64.0)	42.3 (18.0; 71.1)
Hypertension	*n* = 836	*n* = 293	*n* = 478	*n* = 48	*n* = 17
Yes	43.2 (37.7; 48.8)	37.9 (30.1; 46.4)	43.3 (35.8; 51.2)	60.0 (37.8; 78.7)	78.9 (56.0; 91.7)
Cardiovascular disease	*n* = 839	*n* = 294	*n* = 480	*n* = 48	*n* = 17
Yes	18.0 (14.0; 22.8)	20.2 (14.1; 28.1)	15.1 (9.9; 22.2)	28.1 (13.9; 48.6)	34.2 (13.1; 64.3)

CKD: chronic kidney disease; Brazilian NHS: National Health Survey. * Mean and 95% confidence interval.

**Table 3:** Mean and prevalence (%) of consumption frequency of food intake markers in individuals with CKD from the Brazilian NHS, 2013 (*n* = 839).

Food Intake Markers	Prevalence (%) of Consumption Frequency					Mean (95% CI)
	Never	1× or 2× per Week	3× or 4× per Week	5× or 6× per Week	Daily	
Replacement of meals with snacks	67.9	19.7	5.5	2.0	4.8	0.9 (0.7; 1.1)
Sweet sugar beverages	45.7	25.7	10.4	6.0	12.3	1.9 (1.6; 2.1)
Fish	43.0	44.8	9.2	1.6	1.4	1.1 (0.9; 1.2)
Fresh fruit juice	41.2	21.1	13.8	10.6	13.2	2.3 (2.0; 2.6)
Sweets	41.1	27.8	11.3	4.4	15.3	2.1 (1.8; 2.3)
Milk	33.0	10.7	6.4	3.3	46.4	3.8 (3.5; 4.2)
Red meat	14.2	21.4	27.2	12.8	24.5	3.7 (3.4; 3.9)
Fruits	12.4	20.0	18.1	12.9	36.7	4.1 (3.9; 4.4)
Cooked vegetables	12.1	26.1	20.7	12.3	28.9	3.8 (3.5; 4.1)
Raw vegetables	11.6	17.3	19.6	11.3	40.2	4.4 (4.1; 4.6)
Chicken	10.1	40.6	31.5	8.1	9.7	2.8 (2.6; 3.0)
Beans	10.1	12.5	11.3	11.4	54.7	5.0 (4.7; 5.3)
Alcoholic beverages	**0**	**≤1×/month**	**>1×/month**			
	72.0	10.0	18.0			
Excess salt *	**Very high**	**High**	**Appropriate**	**Low**	**Very low**	
	3.5	10.5	48.3	29.8	7.9	

CKD: chronic kidney disease; Brazilian NHS: National Health Survey; 95% CI: 95% confidence interval. * Excess salt consumption: perception of salt intake.

**Table 4:** Factor loadings of food intake markers present in the dietary patterns identified among individuals with CKD from the Brazilian NHS, 2013 (*n* = 839).

Food Intake Markers	Unhealthy Pattern	Healthy Pattern	Communality
Red meat	**0.70**	0.04	0.48
Sweet sugar beverages	**0.54**	0.16	0.31
Alcoholic beverages	**0.41**	0.03	0.17
Sweets	**0.38**	0.05	0.15
Raw vegetables	0.16	**0.76**	0.60
Cooked vegetables	0.06	**0.73**	0.54
Milk	0.09	**0.41**	0.18
Fruits	0.16	**0.63**	0.42
Fresh fruit juice	0.25	**0.43**	0.25
Chicken	**0.47**	0.12	0.23
Fish	**0.49**	0.07	0.25
Excess salt	**0.52**	0.08	0.28
**Variance explained (%)**	18.3	13.8	
**Cumulative variance (%)**	18.3	32.1	
**Eigenvalues**	2.2	1.7	

CKD: chronic kidney disease; Brazilian NHS: National Health Survey. Factor loadings of ≥0.35 and ≤−0.35 are shown in bold for easy reading. Index of Kaiser–Meyer–Olkin (KMO) = 0.66. Bartlett’s test of sphericity (BTS) *p* < 0.001. Extraction model of factors: principal component factors with varimax orthogonal rotation.

**Table 5:** Linear regression models (crude and adjusted) for the association between CKD treatment and mean scores of dietary patterns in individuals with CKD from the Brazilian NHS, 2013 (*n* = 839).

Variable	Unhealthy Pattern		Healthy Pattern	
	Crude β-Coefficient	Adjusted β-Coefficient *	Crude β-Coefficient	Adjusted β-Coefficient *
	(95% CI)	(95% CI)	(95% CI)	(95% CI)
Untreated CKD (*n* = 294) (reference group)	1	1	1	1
Nondialysis dependent (*n* = 480)	−0.28 ** (−0.42; −0.15)	−0.20 ** (−0.33; −0.06)	−0.05 (−0.18; 0.08)	−0.06 (−0.18; 0.06)
Dialysis dependent (*n* = 48)	−0.93 ** (−1.21; −0.65)	−0.80 ** (−1.16; −0.45)	−0.23 (−0.53; 0.06)	−0.17 (−0.40; 0.06)
Renal transplant (*n* = 17)	−0.48 (−1.20; 0.23)	−0.55 (−1.13; 0.03)	0.19 (−0.17; 0.55)	0.32 ** (0.03; 0.62)

CKD: chronic kidney disease; Brazilian HS: National Health Survey; 95% CI: 95% confidence interval. *Adjusted by gender, age, race, education level, location of residence, and Brazilian geographical regions. ** Level of significance of 5%.

## Supplementary Materials

The following are available online at https://www.mdpi.com/2072-6643/11/8/1920/s1, **Figure S1:** Cattel test graph (screen plot).
