# Supplementary figures and images for: Dietary Patterns of Patients with Chronic Kidney Disease: The Influence of Treatment Modality
Source: Nutrients. 2019 Aug 15;11(8):1920. doi: 10.3390/nu11081920 (PMC6723967; doi:10.3390/nu11081920)

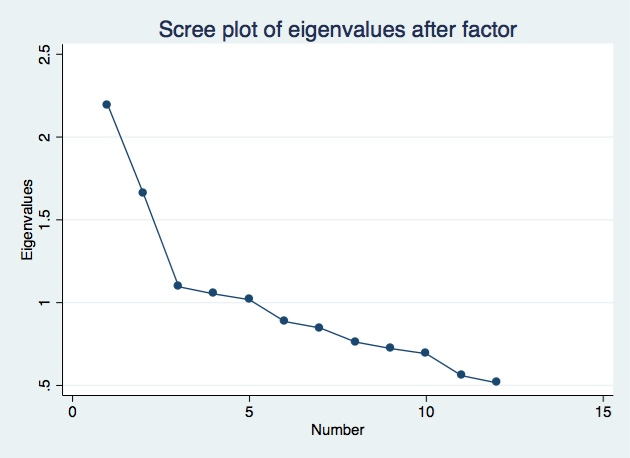

Supplement: Supplementary file 1 [file nutrients-11-01920-s001.zip › nutrients-537698-supplementary.tif]
